# miR Profile of Chronic Right Ventricular Pacing: a Pilot Study in Children with Congenital Complete Atrioventricular Block

**DOI:** 10.1007/s12265-022-10318-w

**Published:** 2022-09-19

**Authors:** Brittany M. Navarre, Katie L. Clouthier, Xuhuai Ji, Anne Taylor, Chad S. Weldy, Anne M. Dubin, Sushma Reddy

**Affiliations:** 1 Department of Pediatrics (Cardiology), Lucile Packard Children’s Hospital, Stanford University, 750 Welch Road, Suite 325, Stanford, CA 94304 USA; 2 Human Immune Monitoring Center and Functional Genomics Facility, Stanford University, Stanford, CA 94305 USA; 3 Department of Medicine (Cardiovascular), Stanford Medical Center, Stanford University, 300 Pasteur Drive, Stanford, CA 94305 USA; 4 Cardiovascular Institute, Stanford University, Stanford, USA

**Keywords:** MicroRNA, Pacing, Children, Remodeling, Heart failure

## Abstract

Chronic ventricular pacing can lead to pacing-induced cardiomyopathy (PICM). Clinical data alone is insufficient to predict who will develop PICM. Our study aimed to evaluate the circulating miR profile associated with chronic right ventricular pacing in children with congenital complete AV block (CCAVB) and to identify candidate miRs for longitudinal monitoring. Clinical data and blood were collected from chronically paced children (*N* = 9) and compared with non-paced controls (*N* = 13). miR microarrays from the buffy coat revealed 488 differentially regulated miRs between groups. Pathway analysis predicted both adaptive and maladaptive miR signaling associated with chronic pacing despite preserved ventricular function. Greater profibrotic signaling (miRs-92a, 130, 27, 29) and sodium and calcium channel dysregulation (let-7) were seen in those paced > 10 years with the most dyregulation seen in a patient with sudden death vs. those paced < 10 years. These miRs may help to identify early adverse remodeling in this population.

**Graphical abstract:**

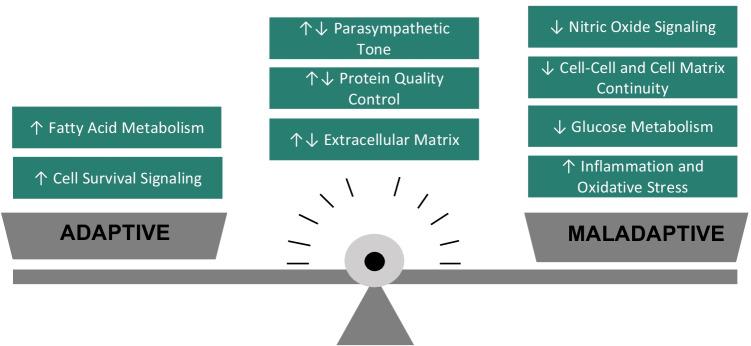

**Supplementary Information:**

The online version contains supplementary material available at 10.1007/s12265-022-10318-w.

## Introduction

Congenital complete atrioventricular block (CCAVB) is estimated to occur in ~ 1 in 15,000 live-born infants [1], and often requires chronic pacing starting at a very young age. Chronic right ventricular pacing produces electromechanical and inter- and intraventricular dyssynchrony which can lead to left ventricular remodeling, dysfunction, and heart failure resulting in pacing-induced cardiomyopathy (PICM) [2–4]. These findings likely occur late in the pathogenesis of PICM when significant adverse myocardial remodeling has already occurred, impacting the effectiveness of strategies such as cardiac resynchronization to improve ventricular function. Indeed, the success of cardiac resynchronization therapy in chronic RV paced children who develop LV dysfunction has shown mixed results [5]. However, there is currently no mechanism to monitor chronically paced patients other than by echocardiography, nor is there a mechanism to monitor for the development of adverse remodeling while ventricular function is still preserved since these patients do not undergo routine myocardial biopsy.

Circulating microRNAs (miRs) are non-coding RNA which regulate gene expression by RNA degradation or translational inhibition [6], and are emerging as key biomarkers of heart failure in cardiomyopathies as well as ischemic and congenital heart disease [7–11]. Marfella et al. discovered a miR profile in chronically paced adult heart failure patients which correlated with clinical improvement after cardiac resynchronization [12]. miRs-26b-5p, 145-5p, 92a-3p, 30e-5p, and 29a-3p were decreased in heart failure but increased after 1 year of cardiac resynchronization therapy, correlating with improved ejection fraction. The objectives of our study were to (i) evaluate the circulating miR profile associated with chronic right ventricular pacing and preserved ventricular function in children with CCAVB, and (ii) identify miRs which may be candidates for longitudinal monitoring.

## Methods

### Study Population

This was a single center, cross-sectional, pilot study of pediatric patients presenting for an electrophysiology outpatient evaluation at a tertiary center between December 2015 and June 2018. The study was approved by the Stanford University Institutional Review Board and assent and consent was obtained from all patients. Inclusion criteria for all patients included age 10–20 years, structurally normal hearts, and normal left ventricular function by echocardiography (left ventricular ejection fraction (LVEF) > 55%). Patients were recruited into two groups — (1) paced group (*N* = 9): patients with a diagnosis of CCAVB and right ventricular pacing for greater than 5 years, and (2) control group (*N* = 13): patients with well-controlled supraventricular tachycardia (atrioventricular node reentrant tachycardia (*N* = 9) or Wolff-Parkinson-White syndrome (*N* = 4)). Patients with hepatic dysfunction, renal dysfunction, pulmonary disease, or febrile illness were excluded due to the potential for these co-morbidities to affect miR expression, clearance, and excretion. Baseline patient demographics and clinical data including New York Heart Association (NYHA) heart failure class were collected. Follow-up data were collected at 3 years. Electrocardiogram and echocardiogram for each patient were performed during a single outpatient visit, at which time a peripheral blood sample was also collected.

### Electrocardiogram and Pacemaker Support

Electrocardiograms (ECGs) performed at the time of a routine outpatient visit were reviewed and the following parameters were recorded — PR interval, QRS duration, and left bundle branch block. Pacemaker information including pacing system, site(s), duration, and percent atrial or ventricular pacing was also obtained. Follow-up data were collected at 3 years.

### Echocardiography

Echocardiograms performed at the time of a routine outpatient visit were reviewed. Data on left ventricular function and size were collected from the reports — LVEF, left ventricular end diastolic dimension in systole (LVEDS) and in diastole (LVEDD), and indexed to body surface area (BSA). Follow-up data were collected at 3 years.

### MicroRNA Expression Profiling and Analysis

Peripheral blood samples were centrifuged then the buffy coat was collected and stored at − 80 °C. Total RNA was isolated from frozen buffy coats (200 μL) using TRIzol (Invitrogen, Carlsbad, CA, USA) and purified using an RNeasy Mini Kit (QIAGEN, Dusseldorf, Germany) according to the manufacturer’s instructions. The expression of some miRs may vary in the different blood fractions (plasma, serum, buffy coat) [13, 14]. The concentration of miRs was the greatest in the buffy coat in our hands based on our prior animal data which showed that the most highly expressed cardiac miR-21 was highly expressed in the buffy coat vs. plasma. Similarly, Glinge et al. have shown that some of the most highly expressed cardiac miRs are highly expressed in the buffy coat vs. plasma [15]. We therefore assessed miR expression in the buffy coat vs. the plasma. RNA quality and quantity were measured using a QIAxpert kit (QIAGEN, Cat. #9,002,340, Germany). Gel electrophoresis was performed on the extracted RNA to ensure sample integrity following which small RNA were quantified using Agilent Bioanalyzer 2100 NANO analysis (Agilent, Santa Clara, USA). Total RNA (100 ng) was labeled with cyanine-3 to generate fluorescent miR, then purified and hybridized onto the SurePrint custom G3 miRNA Microarray (8 × 60 k, p/n G4871A). The slides were scanned and data extracted using Agilent feature extraction (FE) software for miR expression. miR expression analysis was performed using GeneSpring GX 14.9.1 software. Normalized data between the 20th and 100th percentiles with detected probes were used for further analysis. Quality control was performed, following which unpaired *t*-test with Benjamini–Hochberg multiple testing correction was applied to the data. Significantly altered miRs with a corrected *p* value of < 0.05 and with a fold change ≥ 2 up- or downregulated in paced vs. control groups were considered for further analysis. Putative target genes of significantly dysregulated miRs were identified using mirPATH v.7 DIANA tools. Gene Ontology (GO) and pathway analyses were performed to identify important biologic processes, nodal points, and pathways unique to the paced and control groups using Ingenuity Pathway Analysis and Cytoscape software.

### Reverse Transcriptase Polymerase Chain Reaction

The expressions of a subset of dysregulated miRs identified by microarray were validated by Taqman two-step qRT-PCR: upregulated miR-205-3p, miR-210-5p, miR-214-3p, and miR-92a-3p, and downregulated miR-15b-5p, miR-126-5p, miR-130b-5p, miR-148-5p, miR-190a-5p, miR-29a-3p, and miR-27a-3p. 50 ng of RNA was reverse transcribed to cDNA followed by amplification (Applied Biosystems). Ambion mirVana qRT-PCR Primer Sets were used (Supplement Table 1). C. elegans miR-39 was used as the spike in control. Fold change (FC) in expression was assessed between paced vs. control patients using 2 power ΔΔCt method [16].

### Statistical Analysis

Demographic, clinical, ECG, and echocardiogram data are presented as mean ± SEM. An unpaired, 2-tailed Student’s *t*-test was utilized for two-group comparisons of continuous variables with normal distribution and Mann–Whitney test when distribution was not normal. *p* ≤ 0.05 was considered significant.

## Results

### Patient Characteristics

Patient demographics and clinical data for the control and paced groups are shown in Table 1. The mean patient age was 15.03 ± 2 years in the control group and 15.7 ± 2.4 years in the paced group. Forty-six percent were males in the control group and 33% were males in the paced group. All were clinically asymptomatic at the time of sample collection with no hepatic or renal dysfunction and no acute illnesses. Thirty-three percent of the paced group had anti-Rho and anti-La antibodies at the time of diagnosis. Medications included one patient on a beta blocker and another on aspirin in the control group, and one patient on “as needed” albuterol for well-controlled asthma in the paced group. At the time of recruitment, none of the control patients had undergone an ablation or any other surgical procedure. All of the paced patients had undergone a pacemaker implantation and no other surgical procedure. Forty-four percent of the paced patients underwent pacemaker placement in the first week of life, 12% in the first year of life, and the remaining 44% between 5 and 10 years. As expected, the QRS duration was longer in the paced group. Data were collected from transthoracic echocardiograms performed at the time of blood collection (Table 1). LVEF was 63.3 ± 0.01% in controls vs. 60 ± 0.1% in paced patients (*p* = 0.04). All paced patients had dual chamber pacemakers (epicardial systems in 78%, and transvenous systems in 12%), were paced for greater than 5 years (per our inclusion criteria), and had ventricular pacing > 95% of the time (Table 2).

**Table 1:** Demographic and clinical data

	Control group (*N* = 13)	Paced group (*N* = 9)
Age (years)	15.03 ± 0.56	15.70 ± 0.80
Race	Asian/Pacific Islander 15% White 31% Hispanic 31% Black 8% Other 15%	Asian/Pacific Islander 11% White 78% Other 11%
Gender (% male)	46%	33%
Cardiac diagnosis	Typical AVNRT 70% WPW 30%	CCAVB 100%
Prior procedures	None	Pacemaker implantation
Cardiac medications	Beta blocker: 1 patient Aspirin: 1 patient	Albuterol: 1 patient
NYHA class I	100%	100%
QRS duration (msec)	93 ± 4	143 ± 7
LV ejection fraction (%)	63.28 ± 0.01	60.88 ± 0.02
RV fractional area change (%)	47.02 ± 0.02	46.74 ± 0.02

Data are presented as mean ± SEM. *AVNRT*, atrioventricular reentrant tachycardia; *WPW*, Wolff-Parkinson-White; *CCAVB*, congenital complete atrioventricular block; *LV*, left ventricle; *RV*, right ventricle

**Table 2:** Clinical data in chronically paced patients

	Time of sample and clinical data collection (*N* = 9)	Clinical follow-up at ~ 3 years (*N* = 9)
Pacing duration (years)	12.31 ± 1.07	15.05 ± 1.08
Pacing system (% patients)	78% Epicardial dual chamber 22% Transvenous dual chamber	25% Epicardial dual chamber 75% Transvenous dual chamber
Ventricular pacing site (% patients)	22% RV apex 56% RV epicardial 22% LV epicardial	50% RV apex 25% RV mid-septal 12.5% RV epicardial 12.5% LV epicardial
Ventricular pacing (% time)	96% ± 0.02	94% ± 0.03
PR interval (msec)	164 ± 5	149 ± 8
QRS duration (msec)	143 ± 7	140 ± 4
LV ejection fraction (%)	60.88 ± 0.02	58.19 ± 0.02
RV fractional area change (%)	46.74 ± 0.02	45.40 ± 0.02
Clinical status	All NYHA class I	NYHA class I: 8 patients Deceased: 1 patient (VF arrest)

Data are presented as mean ± SEM. *LV*, left ventricle; *RV*, right ventricle

### Unique Circulating miR Expression Profile Is Detected in Chronically Paced Children

**Fig. 1:**
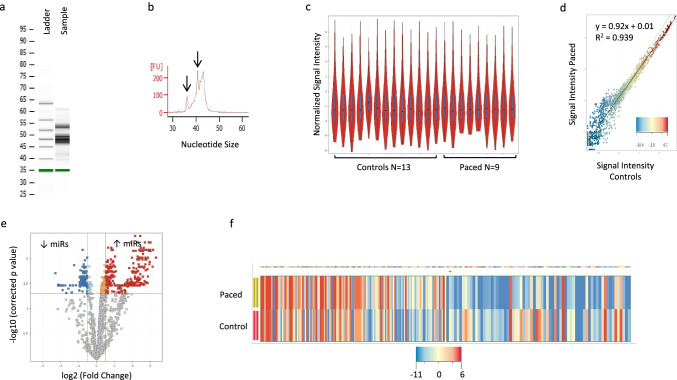
Sample quality control and miR expression in control vs. paced patients. **a** RNA was isolated from the buffy coat and the presence of small RNA with no degradation was confirmed by 2D gel electrophoresis, **b** the presence of small RNAs between 25 and 50 nucleotides including miR (arrows) was confirmed by an electropherogram, **c** violin plot of normalized signal intensity in miR expression among the control (*N* = 13) and paced (*N* = 9) samples demonstrates normal distribution across all samples. **d** Scatter plot demonstrates global miR expression by microarray in controls vs. paced patients. **e**, **f** Volcano plot and heatmap showing significantly up- (red) and downregulated miRs (blue) in paced vs. controls groups by > twofold change in expression and corrected FDR *p* < 0.05. miR, microRNA

miR quality was excellent in all blood samples (Fig. 1a, b). Normalized signal intensity in miR expression among the control (*N* = 13) and paced (*N* = 9) groups demonstrated normal distribution across all samples (Fig. 1c, d). A total of 488 miRs were differentially regulated between the control and paced groups (FC > 2/ <  − 2, corrected *p* < 0.05). 296 miRs were upregulated in the paced group and 192 miRs were downregulated (Fig. 1e, f). To better understand the observed global miRNA expression profile changes, we performed pathway analysis on both upregulated and downregulated miRs.

### miRs Upregulated with Chronic Pacing Predict Poor Protein Quality Control and Cell–Cell Mechanical Uncoupling

**Fig. 2:**
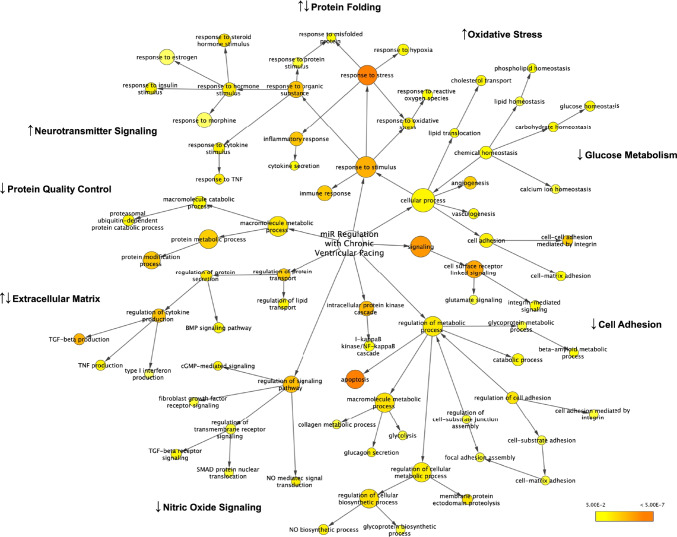
miR regulation with chronic ventricular pacing. Network map highlights significant Gene Ontology (GO) biological processes predicted by the miRs dysregulated in paced patients vs. controls. miRs upregulated with chronic pacing predict downregulation of proteins involved in cell adhesion, glucose metabolism, and protein quality control. miRs downregulated with chronic pacing predict upregulation of oxidative stress and neurotransmitter signaling, with decreased response to estrogen and nitric oxide signaling. miRs dysregulated with chronic pacing also predicted mixed regulation of components involved in protein folding and extracellular matrix. GO biological processes mapped using Cytoscape version 3.8.2. TNF, tumor necrosis factor. TGF, transforming growth factor beta. NO, nitric oxide

Upregulated miRs predicted downregulation in 15 pathways which clustered into 4 groups (Table 3, Fig. 2), (i) structural proteins: O-glycan biosynthesis (protein folding, stability, trafficking to the right cellular location), glycosphingolipid biosynthesis of membranes; (ii) extracellular matrix (ECM): proteoglycans (non-structural components of ECM), adherens junctions which mechanically couple and reinforce cardiomyocytes, Wnt/TGF-β signaling which mediate fibrosis; (iii) metabolism: FoxO signaling pathway which regulates gluconeogenesis and glycogenolysis by insulin signaling and counteracts oxidative stress; and (iv) protein turnover and degradation: lysine degradation which regulates essential amino acid metabolites, ubiquitin proteosomal and lysosomal systems mediating protein quality control, Hippo signaling which is an antiproliferative and cell death pathway.

**Table 3:** Upregulated miRs and downregulated target pathways in chronically paced patients compared with controls

	Downregulated target pathways	Decreased function	miRs enriched in pathways
Upregulated miRs			
Cluster 1 Structural proteins	O-glycan biosynthesis Glycosphingolipid biosynthesis	Protein folding, stability, trafficking to the right cellular location Synthesis of membrane components	miR-513a, 943, 761 miR-761, 449c-5p, 764, 548
Cluster 2 *Extracellular matrix (ECM)*	Proteoglycans in cancer Adherens junctions Wnt/TGF-β signaling	Non-structural component of ECM Cell–cell and cell–matrix continuity Fibrosis	miR-513a, 548, 373p miR-548 miR-513a, 548, 373p, 943, 764
Cluster 3 Metabolism	FoxO signaling pathway	Regulation of gluconeogenesis and glycogenolysis by insulin signaling; counteracting oxidative stress	miR-548, 210
Cluster 4 Protein turnover and degradation	Lysine degradation Ubiquitin proteosomal and lysosomal systems Hippo signaling	Regulation of essential amino acid metabolites Protein quality control Antiproliferative, proapoptotic	miR-548, 373, 205, 499c miR-513a, 548, 514b, 205, 506 miR-513a, 548, 568, 210, 214

*miR*, microRNA; *ECM*, extracellular matrix; *TGF*, transforming growth factor

### miRs Downregulated with Chronic Pacing Predict Altered Parasympathetic Tone as well as Upregulation of TGF-β and Fatty Acid Metabolism

Downregulated miRs predicted upregulation in 16 pathways which clustered into 4 groups (Table 4, Fig. 2), (i) O-glycan biosynthesis; (ii) extracellular matrix: profibrotic TGF-β signaling, ECM-receptor interaction which includes the crosstalk between ECM structural proteins such as collagens, laminin and fibronectin, and cells via transmembrane proteins such as integrins and proteoglycans; (iii) fatty acid biosynthesis; and (iv) neurotransmitter signaling enhancing both vagal nerve inhibition via GABA, vagal nerve activation via muscarinic receptors, and inhibition of estrogen signaling which impairs nitric oxide metabolism and vasodilation, L-type Ca and K channel regulation, and intracellular signal transduction pathways. In summary, components of structural and extracellular matrix are both up- and downregulated simultaneously in chronically paced patients while there is preserved fatty acid metabolism and impaired vascular signaling.

**Table 4:** Downregulated miRs and upregulated target pathways in chronically paced patients compared with controls

	Upregulated target pathway	Increased function	miRs enriched in pathways
Downregulated miRs			
Cluster 1 Structural proteins	O-glycan biosynthesis	Protein folding, stability, trafficking to the right cellular location	miR-195-5p, 126-5p, 182-5p
Cluster 2 Extracellular matrix (ECM)	Proteoglycans in cancer TGF-β signaling pathway ECM-receptor interaction, ARVC	Non-structural component of ECM Profibrotic Crosstalk between ECM and cells via transmembrane receptors Dysregulation of cell adhesion	miR-195-5p, 126-5p, 182-5p miR-195-5p, 374a-5p, 942-5p, 26a-5p, 130b miR-182-5p, 942-5p, 32-5p, 659-5p miR-942-5p, 148b-5p, 589-3p
Cluster 3 Metabolism	Fatty acid metabolism	Fatty acid biosynthesis and degradation	miR-195-5p, 545-5p, 374a-5p 589-3p, 15b-3p
Cluster 4 Hormonal and neurotransmitter signaling	GABAnergic synapse; morphine addiction Inhibition of estrogen signaling	Inhibit central vagus nerve outflow; Activate parasympathetic discharge via muscarinic receptors Unfavorable lipid profile, decreased NO metabolism — impaired coronary vasodilation, L-type Ca, and K channel regulation	miR-374a-5p, 126-5p, 182-5p, 148b-5p, 183-5p miR-126-5p, 182-5p, 454-3p, 215-5p

*miR*, microRNA; *ECM*, extracellular matrix; *TGF*, transforming growth factor; *ARVC*, arrhythmogenic right ventricular cardiomyopathy; *GABA*, gamma-aminobutyric acid; *NO*, nitric oxide; *Ca*, calcium; *K*, potassium

### Unique miR signature seen Based on Duration of Pacing

**Fig. 3:**
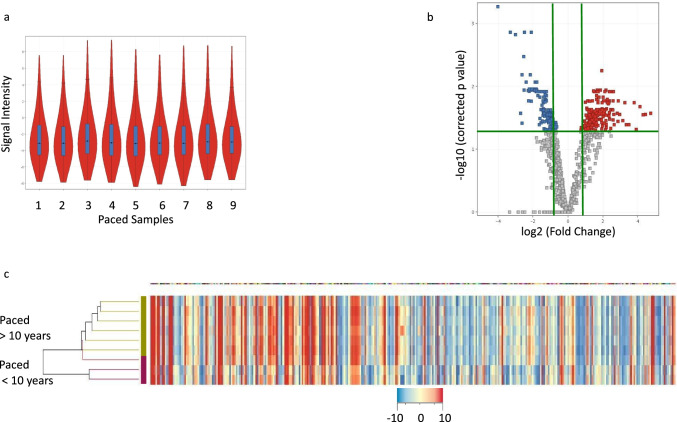
miR expression in paced patients by duration of pacing. **a** Violin plot of normalized signal intensity in miR expression among all the paced samples demonstrates normal distribution. **b**, **c** Volcano plot and heatmap showing significantly up- (red) and downregulated miRs (blue) in patients paced > 10 years vs. patients paced < 10 years by > twofold change in expression and corrected FDR *p* < 0.05. miR, microRNA

**Fig. 4:**
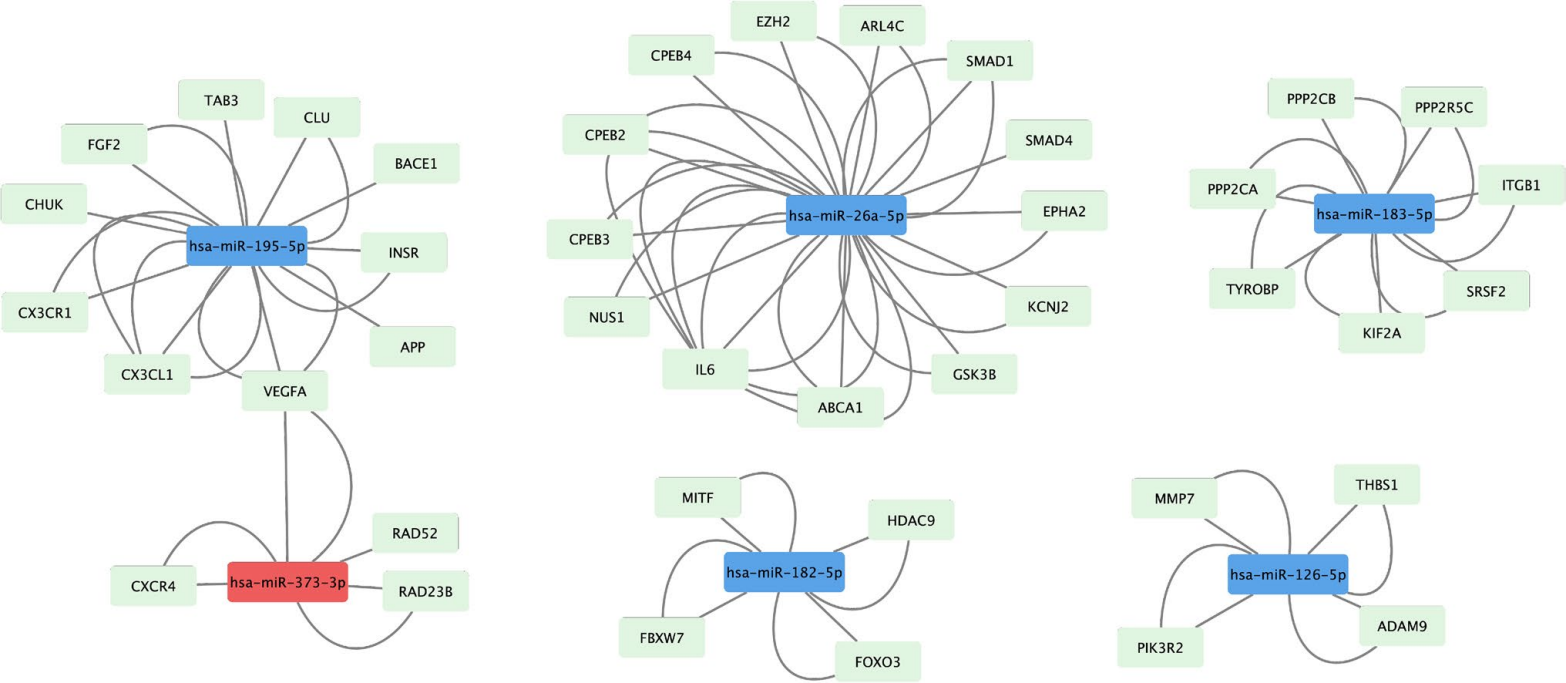
Target proteins of select dysregulated miRs secondary to chronic pacing. Bioinformatically predicted target genes of dysregulated miRs secondary to chronic pacing were mapped using Cytoscape version 3.8.2. Downregulations of miR-195-5p and miR-26a-5p predict upregulation in extracellular matrix proteins; downregulation in miR-183-5p predicts upregulation in protein phosphatases and integrins; downregulation in miR-182-5p predicts upregulation in vascular inflammation via HDAC9 and MITF; and downregulation in miR-126-5p predicts upregulation in cell–cell interaction via ADAM9 and extracellular matrix via MMP7 and THBS1. Upregulation of miR-373-3p predicts downregulation of target genes involved in DNA repair and synthesis of transmembrane receptors. Red, upregulated miR; blue, downregulated miR; green, target proteins

**Fig. 5:**
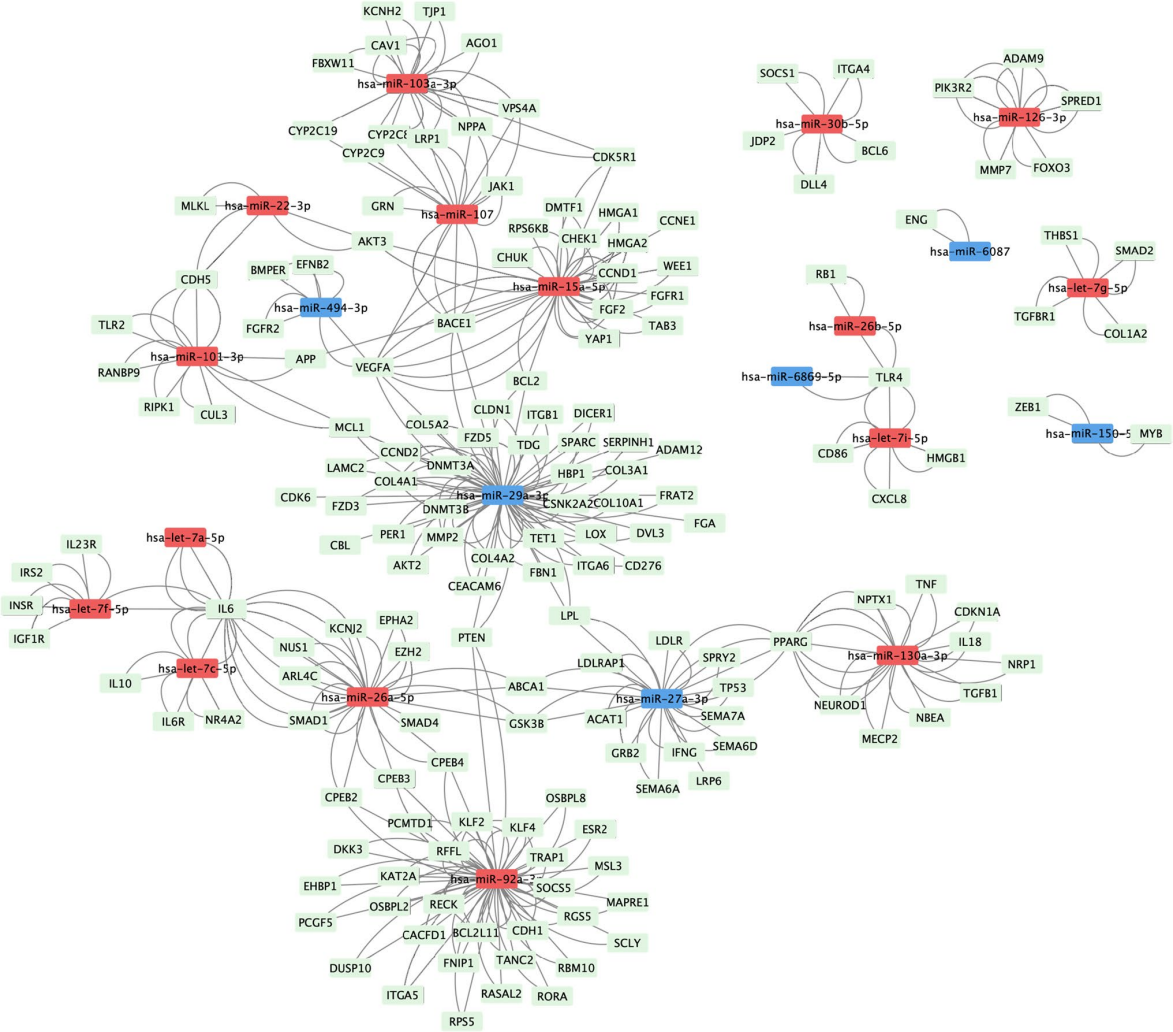
Target proteins of dysregulated miRs in the deceased patient compared to patients paced for < 10 years. Bioinformatically predicted target genes of dysregulated miRs secondary to chronic pacing were mapped using Cytoscape version 3.8.2. Upregulation of let-7 predicts downregulation of proteins involved in myocardial sodium and calcium channel homeostasis. Upregulations of miR-92a and miR-130 predict downregulation of proteins important for maintaining vascular endothelial health and preventing fibrosis. Downregulations of miR-29 and miR-27 predict upregulation of profibrotic signaling. Red, upregulated miR; blue, downregulated miR; green, target proteins

We next evaluated whether patient demographics and the duration of pacing influenced the circulating miR signature. The circulating miR signature was not influenced by patient age, gender, or race. Interestingly, with longer duration of pacing 14.17 ± 0.7 years (*N* = 6) vs. 8.49 ± 0.6 years (*N* = 3) (*p* = 0.001), 194 miRs were upregulated and 97 miRs were downregulated (Fig. 3). Age at the time of sample collection was similar between the two groups (15.6 ± 1.1 vs. 15.9 ± 1.5 years, *p* = 0.87). The longer duration of pacing group had pacemakers placed between 0 and 1 year of age (with one exception), while the < 10 year paced group had pacemakers placed at > 5 years of age. Upregulated miRs predicted downregulation of target pathways involved in focal adhesion via cadherins and protocadherins (miR-548, miR-449c-5p), protein phosphorylation, ATP binding, kinase activity (miR-758, miR-183), and protein quality control via protein ubiquitination, conjugation, and ligase activity (miR-513a, 548, 514b) (Table 5). Downregulated miRs predicted upregulation of target pathways involved in extracellular matrix (miR-195-5p, 374a-5p, 942-5p), inflammation (miR-126-5p, miR-199a, miR-182-5p), and zinc finger protein family of transcription factors (miR-181a, miR-26a, miR-148b, miR-15b, miR-374a) (Table 5). Predicted target proteins of select dysregulated miRs are shown in Fig. 4. Among the patients with longer duration of pacing, one patient died following a ventricular fibrillation induced cardiac arrest. Clinical characteristics of this patient were similar to the remainder of the chronically paced group at the time of sample collection and at 3-year follow-up. However, the miR signature differed. Upregulated miRs predicted downregulation of pathways involved in myocardial sodium and calcium channel homeostasis (let-7), as well as pathways important for maintaining vascular endothelial health and preventing fibrosis (miR-92a, miR-130). Downregulated miRs predicted upregulation of profibrotic signaling (miR-29, miR-27) (Fig. 5).

**Table 5:** Predicted target pathways of miRs dysregulated with longer duration of pacing

Cluster	Enrichment score	Cluster category	Benjamini–Hochberg corrected *p* value
Upregulated miRs predict downregulation in target pathways			
1	6.02	Cell and membrane adhesion, cadherins	2.70E-02
2	4.8	Protein phosphorylation, ATP binding, kinase activity	5.50E-06
3	3.81	Protein ubiquitination, conjugation, ligase activity	1.60E-05
4	3.66	Focal adhesion, protocadherin	1.40E-04
5	3.54	Ras nucleotide exchange factors, transcription regulation	1.70E-02
6	3.43	Microtubule binding	3.80E-02
Downregulated miRs predict upregulation in target pathways			
1	1.5	Extracellular matrix, transmembrane proteins	3.10E-02
2	1.11	Inflammation	3.90E-02
3	1.04	Transcription factors	4.20E-02
4	1.02	Inflammation serine peptidase inhibitors	4.10E-02
5	0.65	Homeobox	5.20E-02
6	0.44	Zinc finger protein family of transcription factors	5.40E-02

*miR *- microRNA

### qPCR Validation of miR Microarray Data

**Fig. 6:**
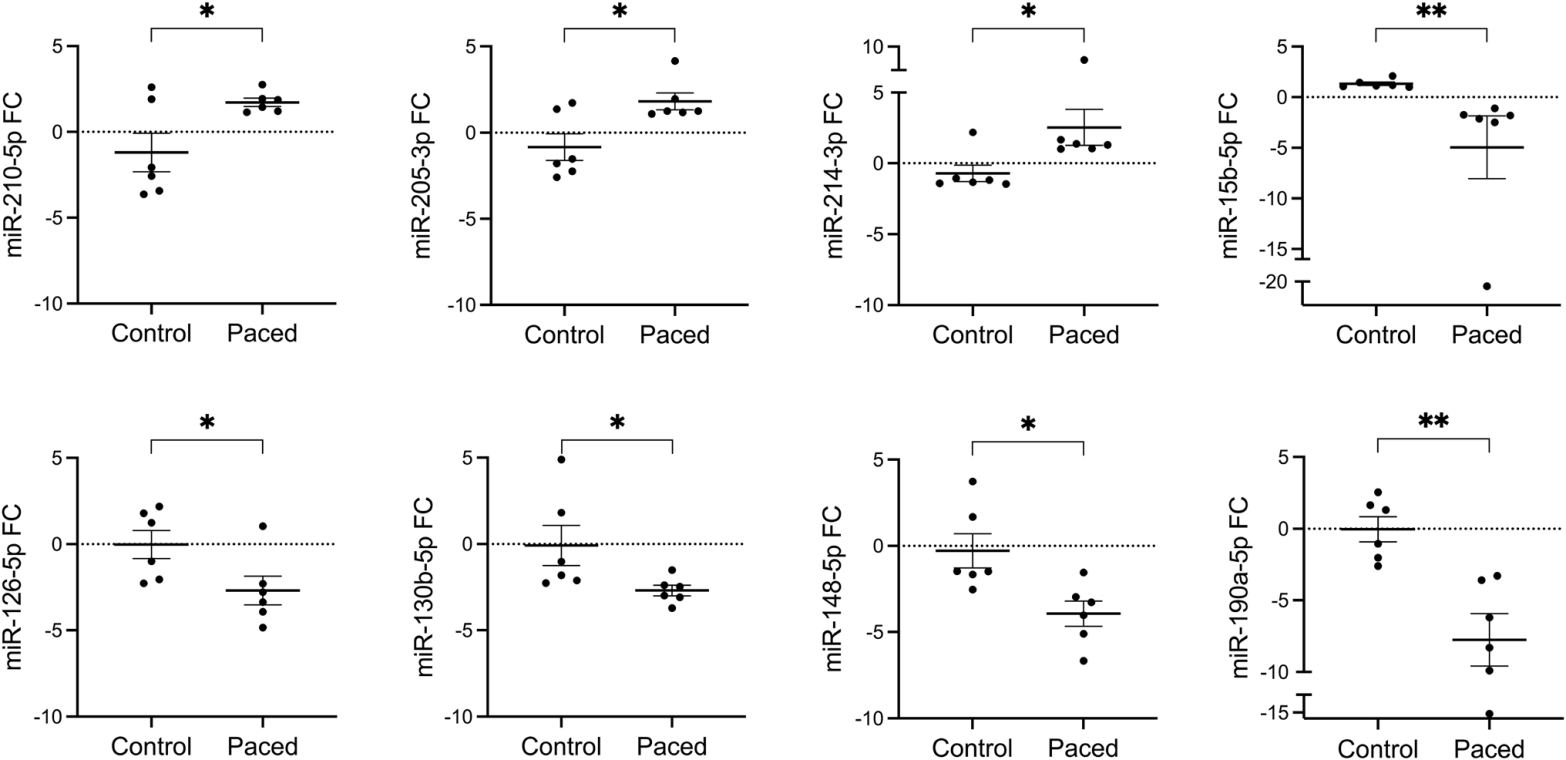
qPCR validation of miRs. Select miRs known to be dysregulated in adult heart failure were validated using qPCR. qPCR expression of the miRs in control vs. paced patients (*N* = 6/group) correlated with miR expression via microarray. We tested three upregulated miRs: miR-214-3p, miR-210-5p, and miR-205-5p and five downregulated miRs: miR-130b-5p, miR-190a-5p, miR-148b-5p, miR-126-5p, and miR-15b-3p. Data are represented as mean and standard error of mean. Unpaired, Student’s *t*-test was used where distribution was normal (miR-205, 210, 126, and 148) and Mann–Whitney test was used where distribution was not normal (miR-214, 15, 130, 190). *p* ≤ 0.05 (✱); *p* ≤ 0.001 (✱✱). miR, microRNA; FC, fold change

qPCR validation of miR microarray results was performed and confirmed on 3 select upregulated miRs: miR-205-5p, miR-210-5p, and miR-214-3p, and on 5 select downregulated miRs: miR-15b-3p, miR-126-5p, miR-130b-5p, miR-148b-5p, and miR-190a-5p (Fig. 6). These particular miRs were selected for qPCR validation based on (i) their high expression, (ii) being upregulated (miR-205-5p, miR-210-5p, miR-214-3p, Table 3) or downregulated (miR-15b-3p, miR-126-5p, miR-130b-5p, miR-148b-5p, miR-190a-5p, Table 4) with chronic pacing, and (iii) previous studies showing their high expression and roles in regulating key biologic processes in other forms of heart failure [17–23]. Interestingly, miRs reported as having favorable prognostic value when downregulated in adult heart failure were among the most downregulated in children who were paced for < 10 years compared with children paced for > 10 years, namely miR-15b-5p (FC-7), miR-148b-5p (FC-7), and miR-190a-5p (FC-15). Additional qPCR validation was performed for expression of select miRs which were further dysregulated in the deceased patient when compared to the rest of the chronically paced group. Consistent with the microarray data, there was significant downregulation of miR-29a-3p (FC-2.2) and miR-27a-3p (FC-2.5), as well as upregulation of miR-92a-3p (FC-3.3).

## Discussion

Chronic ventricular pacing in children with CCAVB and normal cardiac structure can lead to pacing-induced cardiomyopathy in 5–10% of patients. This pilot study in children with CCAVB evaluated the circulating miR signature to understand the response to chronic ventricular pacing. We identified both adaptive and maladaptive miR signaling at a time when ventricular function is still preserved with a shift toward more maladaptive signaling with longer duration of pacing. Based on the circulating miR dysregulation, fatty acid metabolism and cell survival were upregulated, while nitric oxide signaling and cell–cell and cell–matrix continuity were downregulated and inflammation and oxidative stress were upregulated. We also show a subset of dysregulated miRs which could be implicated in the development of an arrhythmogenic substrate with chronic pacing.

Similar to other etiologies of heart failure, impaired fatty acid oxidation has been reported in animal models of pacing-induced cardiomyopathy [24–28]. In contrast, we observed downregulation of miR-195 which predicts heightened fatty acid biosynthesis, highlighting the ongoing ability to meet the increased demands of the stressed heart. A transition to upregulation of miR-195 may predict the development of heart failure and could be used as a tool to follow disease progression [29]. Adaptive signaling was also predicted by preservation of pathways involving crosstalk between ECM and transmembrane proteins, as well as maintenance of structural integrity, and cell survival and function [30].

Maladaptive miR signaling associated with chronic ventricular pacing was characterized by an upregulation in the miR-548 family of miRs, which are known to inhibit cell–cell and cell–matrix continuity as well as protein quality control, and may therefore induce electromechanical uncoupling. Chronic right ventricular pacing is known to cause electromechanical uncoupling [31–34], which over time may lead to ventricular dysfunction and arrhythmias [3, 35]. While heightened oxidative stress and inflammation have been demonstrated in animal models of chronic ventricular pacing [36, 37], we show here for the first time that this adverse myocardial remodeling may be reflected in the circulating miR signature, thereby raising the possibility of its utility in following disease progression noninvasively. [36, 38]. The miR signature in our chronically paced patients also implicates impaired proteosomal-ubiquitin systems which maintain protein quality and prevent accumulation of damaged proteins such as quality control of gap junction proteins [39–41].

It is unclear whether the duration of pacing is an independent risk factor for the development of PICM. Long-term follow-up studies suggest that chronic pacing can result in significant histopathological changes but the overall incidence of LV dysfunction remains quite low over time [3, 42–44]. We show a miR signature predicting ongoing maladaptive signaling and reduced adaptive signaling with longer duration of pacing despite preserved ventricular function. Downregulations of miR15b-3p and miR 190a-5p have been associated with preservation of cardiac function in adult patients with heart disease [17, 18]. Our data is consistent with this. However, patients paced > 10 years showed an upregulation in these miRs compared to those paced < 10 years suggesting a preclinical shift away from preserved ventricular function. Patients paced for > 10 years also showed downregulation of miR-126 supporting vascular endothelial dysfunction and heightened inflammation with longer pacing duration [19]. Interestingly, one patient who was paced for > 10 years had a miR profile predicting dysregulation of myocardial sodium and calcium channels (let-7), endothelial dysfunction (miR-92a), and fibrosis (miR-27, miR-29, miR-130). This arrhymogenic and profibrotic substrate was out of proportion to the remainder of the chronically paced group. This patient experienced an unexplained terminal ventricular arrhythmia 3 years later. These findings are consistent with studies demonstrating that fibrosis and myocardial ion channel dysregulation provide a mechanistic substrate for ventricular arrhythmias across multiple etiologies of heart failure [45, 46]. Taken together, these findings suggest that the let-7 family as well as miRs-92a, 130, 27 and 29, which have shown promise as candidate biomarkers in other forms of heart failure, may also be helpful for predicting morbidity in chronically paced children [47–51].

The present study does not elucidate whether the circulating miR signature we identified in paced patients with CCAVB originates from the stressed myocardium itself, affected target organs, or represents a larger systemic response to myocardial stress. Additionally, we cannot eliminate the possibility that this miR signature could reflect other sources of myocardial stress such as the initial immunologic insult caused by anti-SSA/SSB antibodies—the cause of CCAVB in our study population. However, studies of anti-SSA/SSB induced cardiomyopathy suggest that patients generally present earlier in life and it is additionally characterized by endomyocardial fibroelastosis and decline in cardiac function which was not seen in our patients [52, 53]. In addition, while we show enhanced miR dysregulation with longer pacing duration, we cannot elimitate the potential influences of age at the time of pacemaker placement, gender, or ethnicity on miR regulation. Lastly, it is not yet known whether the adverse remodeling pathways identified in this study are those which ultimately cause PICM.

## Conclusions

In summary, we identified a unique, noninvasive, circulating miR signature in chronically paced children with normal function predicting a balance of adaptative and maladaptive signaling which may be important for preserving ventricular function. Longer duration of pacing (> 10 years) was associated with ongoing maladaptive signaling as well as a dampening of adaptive signaling when compared to those with shorter pacing duration (< 10 years). Given that ventricular function was maintained in both subgroups, this difference could signify that ongoing remodeling is occurring but is not yet sufficient to cause measurable changes in ventricular function. miR-15b, miR-126, and miR-130, which were significantly dysregulated with longer pacing duration in the present study and known to be dysregulated in other forms of heart failure, may be good candidates for following this disease progression. Finally, miR-29a, miR-27a, and miR-92a, which predicted enhanced ion channel dysregulation and fibrosis and were further dysregulated in the chronically paced patient who suffered a terminal ventricular arrhythmia, may be important for predicting risk for later morbidity.

## Supplementary Information

Below is the link to the electronic supplementary material.

Supplementary file1 (PDF 34 KB)

## Data Availability

The data generated and analyzed during the current study are available from the corresponding author on reasonable request.

## Abbreviations

PICM: Pacing-induced cardiomyopathy
miR(s): MicroRNA(s)
CCAVB: Congenital complete atrioventricular block
LVEF: Left ventricular ejection fraction
LVEDS: Left ventricular end diastolic dimension in systole
LVEDD: Left ventricular end diastolic dimension in diastole
RV FAC: Right ventricular fractional area change
BSA: Body surface area
FC: Fold change
ECM: Extracellular matrix
PBMCs: Peripheral blood mononuclear cells
