# Supplementary material for: miR Profile of Chronic Right Ventricular Pacing: a Pilot Study in Children with Congenital Complete Atrioventricular Block
Source: J Cardiovasc Transl Res. 2022 Sep 19;16(2):287–99. doi: 10.1007/s12265-022-10318-w (PMC10151311; doi:10.1007/s12265-022-10318-w)
Supplement: Supplementary file 1 — Supplementary file1 (PDF 34 KB) [file 12265_2022_10318_MOESM1_ESM.pdf]

Supplement

Supplement Table 1. microRNA primer sequences used for RT-PCR

microRNA	Mature Sequence
hsa-miR-205	UCCUUCAUUCCACCGGAGUCUG
hsa-miR-210-5p	CUGUGCGUGUGACAGCGGCUGA
hsa-miR-214-3p	ACAGCAGGCACAGACAGGCAGU
hsa-miR-15b	CGAAUCAUUAUUUGCUGCUCUA
hsa-miR-126	UCGUACCGUGAGUAAUAAUGCG
hsa-miR-130b-5p	ACUCUUUCCCUGUUGCACUAC
hsa-miR-148-5p	AAAGUUCUGAGACACUCCGACU
hsa-miR-190a-5p	UGAUAUGUUUGAUAUAUUAGGU
